# “The individual at the centre” – a grounded theory explaining how sport clubs retain young adults

**DOI:** 10.1080/17482631.2017.1361782

**Published:** 2017-08-30

**Authors:** Eva-Carin Lindgren, Claes Annerstedt, John Dohsten

**Affiliations:** ^a^ School of Health and Welfare, Halmstad University, Halmstad, Sweden; ^b^ Department of Food and Nutrition, and Sport Science, University of Gothenburg, Gothenburg, Sweden

**Keywords:** grounded theory, intersectionality, participation, social connectedness, young adults

## Abstract

**Purpose:** There is still a lack of knowledge regarding which social processes occur in sport clubs and what factors influence young adults to want to remain in a sport club context. Thus, the purpose of this study was to construct a grounded theory (GT) explaining how sport clubs can retain their young adults. **Method:** The study uses an intersectional approach. In line with constructivist GT methodology, data from 14 focus-group interviews (27 coaches and 28 young adults) were collected and analysed using a constant comparative method. **Results:** The core category, “The individual at the centre of a community”, summarizes a process, whereby the generated GT contains three main categories, namely (1) “Participation and influence”, (2) “Social connectedness” and (3) “Good conditions”. **Conclusions:** The coaches put the individual at the centre of a community and pay attention to the needs and interests of all the young adults, regardless of their background, ambitions, and skills. However, while the idea of a moral imperative to provide for diversity was not directly absent in the discussions with both the coaches and young adults, most of the diversity approaches seemed to be based on ambition and skills, gender, age and sexuality.

Sport clubs are regarded as a suitable setting to promote participation in physical activity, and participating in sports contributes to different physiological, psychological and social health benefits. Furthermore, sport clubs have been found to provide additional benefits that are not obtained in other leisure-time exercises (Eime, Young, Harvey, Charity, & Payne, 2013). Despite these potential advantages, not all participants experience sports positively. There have been indications of reduced participation in sports among the young people of today compared to a decade ago. The decline in youth sports activity is highest among older adolescents, specifically among girls and young women, and the decrease in participation rate cannot be explained by changes in age population only (Norberg, 2015). Statistics also show that youth sports activity levels are lower in socioeconomically weak environments compared to socioeconomically strong areas (Norberg, 2015). Moreover, these dropout rates have not increased only among young people in Sweden. The trend is also evident in other countries (for example, Rottensteiner, Laakso, Pihlaja, & Konttinen, 2013). In a systematic review of dropouts from organized sports among children and youth, findings indicated that intrapersonal and interpersonal constraints were more frequently associated with dropping out of sports than structural constraints. However, no simple explanations for this tendency were given, as many factors interact with each other (Crane & Temple, 2015). Physical activity levels and determinants were studied by Zimmermann-Sloutskis, Wanner, Zimmermann, and Martin (2010). The most important result in their study was the strong effects of sport club membership on general physical activity, as non-membership was the strongest predictor of inactivity among participants.

**Table 1.** Focus-group interviews with coaches and young adults in various sport clubs allocated by sport and gender.

	Coaches		Young adults	
Sport	Women	Men	Women	Men
Archery	2	2	1	2
Thai boxing	1	3	2	2
Boxing	1	3	1	3
Floorball	2	2	3	3
Equestrian	3	0	3	0
Orienteering	1	3	2	2
Swimming	1	3	2	2
Total	11	16	14	14

In order to keep more young adults physically active within sport clubs, researchers state that these organizations must adapt their activities and content to the needs of young people (Thedin Jacobsson, Lundvall, Redelius, & Engström, 2012). Every year, the Swedish government gives extensive financial support to the Swedish Sports Confederation (SSC) in order to organize sports, i.e., to retain as many participants as possible (Norberg, 2015).

Studies that focus on what is keeping young adults in sports mention, for example, that the activities need to be fun, among friends, allow participants to learn new things, competitive, and make a contribution to improved health and well-being (i.e., Goncalves, Carvalho, & Light, 2011; Light, Harvey, & Memmert, 2013; Lindgren, 2002; Thedin Jakobsson, 2014; Thedin Jakobsson, Lundvall, & Redelius, 2014; Ullrich-French & Smith, 2009). Previous research indicates a gap regarding the circumstances that encourage young people to remain at their sport clubs. Studies have to some extent addressed the issue and what has emerged is that the social and cultural contexts children and young people are in and which exist in their clubs are important for explaining why they are still involved in sport clubs (Larsson, 2013; Light et al., 2013; Lin, Chalip., & Green, 2016; Trondman, 2005; Wagnsson, 2009).

Kokko, Green, and Kannas (2013) state that sport clubs remain underdeveloped and underutilize settings for health promotion, and that cultural, social, economic and environmental determinants within sport clubs support a change in favour of health promotion. They argue that retaining young people at sport clubs could form part of a health-promotion approach. In other words, when analysing the conditions of young adults in the sport clubs and how the needs of these individuals can be met, the role of the coaches becomes crucial as they operate and interact with young adults daily. Likewise, the general orientation of sport clubs and the official guidance from coaches are crucial when trying to retain young people in sport clubs. However, there is still a lack of knowledge regarding which social processes occur in sport clubs and what factors influence young adults to want to remain in a sport club context.

Understanding how factors like these relate to each other will help us see beyond the liberal scientific framework to further emphasize a more democratic access to institutionalized sports for young adults. Thus, the purpose of this study was to construct a grounded theory (GT) explaining how sport clubs can retain their young adults. We specifically aim to answer the following research questions: what is the main issue concerning diversity among young adults in order to continue in their sport clubs? What are the main issues for coaches regarding retaining a diverse population of young adults? Through this study, we aim to explore the social processes concerning the methods that sport clubs, specifically the coaches, engage in to encourage young adults to continue participating at sport clubs, regardless of their background. To get a more nuanced picture, it is important to listen to both young adults and their coaches.

## An intersectional framework

When the focus is on young adults in general, and not just a certain group, intersectionality is a useful framework. Intersectionality is the idea that multiple identities and conditions intersect to create a whole that is different from the separate identities. Such a framework can help to highlight how sports have taken into account that young adults have different identities and needs and, thus, identify themselves with different groups. It means seeing young adults from different perspectives, and understanding that their various affiliations or positions—for example, on the basis of gender, sexuality, ethnicity, class, age, disability—influence their identities, which have consequences regarding to what extent they want to and can remain in their sports. For some, it might be easier to stay at their sport clubs, while for others it might be much harder, both individually and structurally. Thus, intersectionality might assist in visualizing different conditions that determine the opportunities and space given to young adults, which affect their feelings regarding who they are, what they can accomplish, and what opportunities are available to practise sports at their sport clubs (Mattsson, 2010).

When an intersectional perspective analyses experiences, identities and opportunities based on a variety of positions, it fosters a multidimensional understanding of power. Power is seen as being exercised by the people through the process of creating inequality in specific contexts (de los Reyes & Mulinari, 2005). To analyse the power, therefore, means to visualize the standards that govern how sport clubs retain members, and who and what these standards influence, for example, offered activities, factors that encourage young adults to remain at their sport clubs. Scholars have shown that, in practice, sport clubs often emphasize one side of diversity over others in ways that often overlook or ignore the intersections between gender, culture, (dis)ability, and racial/ethnic inequalities (Spaaij et al., 2014).

## Methodology

The constructivist grounded theory (CGT) of constant comparison of data, reflexive memoing, theoretical sensitivity, and theoretical sampling used in this study were based on those described by Charmaz (2006). This approach brought subjectivity into view and assumes that people, including researchers, are constructing the realities in which they participate. Constructivist grounded theorists aim for abstract understanding of studied life and view these analyses as located in time, place, and the situation of inquiry (Charmaz, 2006). Therefore, the findings of the study are, at all times, provisional and specific to the contexts in which they were developed. The first authors of this study have employed GT methodology in prior work.

### Sampling and participants

Adhering to our aim to generate substantive theory, contextual specificity was sustained by sampling both coaches and young adults from sport clubs that have managed to retain many adolescents and young adults in their activities. Due to the need to consider diverse perspectives, the research was engaged with a broad intention to work toward a final sample of sport clubs that have managed to retain individuals in the 18–25 age range who were not mainly active elite athletes, but rather were active at a lower level. The study also aimed to obtain a final sample that had represented different sports, socioeconomic differences among members, diversity, and locations within both sparsely populated and urban areas.

As CGT is an iterative research process, in which there is a repeated interaction procedure between data collection and analyses, theoretical sampling was employed (Charmaz, 2006). The full sampling and data-collection process occurred over three phases. During the first phase, the Sport Federation of Västra Götaland supported the identification of potential sport clubs in which many adolescents and young adults participated in their activities. Prior to the focus-group interviews (FGs) with representatives at the clubs, the individuals were required to answer the question regarding whether they were “good at retaining youth and young adults at their sport club”. Of the identified (*N* = 10) and surveyed sport clubs, two clarified that many adolescents took part in activities at their organizations, but not a lot of young adults were active. During a focus-group interview at another club, we discovered that this entity also did not have many young adults as members, only children and adolescents. Therefore, these three clubs were withdrawn from the study.

In the next phase, coaches (both women and men) who trained young adults were selected. During the third phase, young adults (both women and men) who were in the 18–25 age range were selected for the study. Recruitment took place from October 2014 to June 2015. Three of the sport clubs represented the most popular sports for children and adolescents in Sweden: floorball, equestrian, and swimming (Norberg, 2015). The overall sample included seven sport clubs, consisting of 14 focus groups with a total of 27 coaches and 28 young adults (Table I). As the data from these coaches and young adults provided unique perspectives on previously identified concepts and categories, but no fundamentally distinct insights, saturation was deemed to have been achieved at this point (Corbin & Strauss, 2008). Confidentiality was assured and all participants provided both oral and written informed consent. The study was approved by the Regional Ethical Review Board of the University of Gothenburg (EPN 583–14).

### Focus-group interviews

FGs were employed as they foster social interaction as individuals discuss a specific phenomenon amongst themselves and because the format allowed maximum inter-participant interaction. Before meeting with the main participants, the focus, content, and clarity of an initial interview guide had been piloted with two FGs (coaches and young adults). While the core questions of the interview guide were not altered by this pilot work, follow-up probes were modified to allow for detailed discussion on these particular factors. An interview guide was included as a support for the FGs to capture the details regarding the perspective of retaining adolescents and young adults at their sport clubs that may not have previously been covered. As part of the FGs, preliminary background questions about the mission of the coaches at the sport clubs were included. The following key questions were included in the interview guide with the coaches. What core values are important to order to retain the young adults at your sport club? What do you do in a concrete way to keep your young adults within your sport clubs? How do you take into account young adults’ wishes? How do you take advantage of young adults’ competences within your sport clubs? What could your sport club improve, so that even more young adults would want to and be able to stay at the club? For the young adults, the following key questions were asked. What makes you keep participating in sport at your sport club? What makes your sport at your sport club so interesting? What do the sport club and, particularly, the coaches do in order to keep young adults in your sport club? Which individuals are welcome to join you in your sport club? What are your possibilities of being involved in and influencing the activities in your sport club? What are your experiences that your wishes and expectations are accounted for, in your sport club? What could promote a sound development in your sport club to keep further young adults staying at the club?

All FGs were recorded using a portable audio recorder (Zoom H4n). At the beginning of each FG, participants were told that all statements would remain within the group, that there were no right or wrong answers, and that all thoughts and opinions were important. However, the last four FGs (of 14) were not transcribed. The reason for this was that a tentative theory had gradually evolved and was utilized in the final FGs in order to further confirm and develop the theory. In these cases, the third author listened to the participant audio files, wrote reflexive notes about the emerging concepts, summed up the content of the FGs, and discussed the reflections with the first author.

In 11 of the FGs, four participants attended each group, and in three of the FGs three participants attended. All the groups were facilitated by the third author, and the same procedures were implemented for each group. A person from the Sport Federation of Västra Götaland observed 10 of the FGs, which was regarded as important for the analysis of the interaction in the FGs. All interviews were conducted at locations chosen by the participants, and lasted between 65 and 100 min. After each FG, the interviews were analysed by the research manager and first author (ECL); the results of this analysis guided the subsequent data production.

### Data analysis

In line with CGT procedures, data analysis began after the first FG and continued in an iterative manner until theoretical saturation was reached after 14 FGs, when further data generation elicited no new theoretical insights around key patterns in the data, and the relationship among categories was well established (Charmaz, 2006). The data were analysed using *initial and focused coding*, *analytic reflexive memos*, and *theoretical sampling* (Charmaz, 2006).

*Initial coding* was performed via an initial line-by-line coding on the FGs transcripts. A code labelled “the individual at the centre” was generated as a general category, demarcating any comments referring to sport clubs and the way the coaches managed the concepts that affect young adults. From this main category, 25 tentative subcategories emerged.

*Focused coding* was used to delve into the tentative subcategories in a comparative and iterative process to explore the relationships between concepts. Six subcategories and three main categories emerged (Charmaz, 2006).

*Analytic reflexive memos* were written throughout this research process, consisting of theoretical notes about the data and their conceptual connections (Charmaz, 2006). In memoing, the recorded ideas about codes and the relationships among them, (sub)categories, and properties were constructed by constantly questioning data and making the connection between what was discovered in the data and what we knew and had experienced. Memo-writing and the collaboration between the first and the second authors helped ensure that the analysis was driven by the data, and not by the researchers.

*Theoretical sampling* was used to deepen the analysis, and to develop and refine the categories in the emerging theory (Charmaz, 2006). Thus, we tested new ideas to see how well these concepts fitted the collected data by constantly checking and re-checking. An example of this was when the coaches described how they had worked to retain young adults in their sport clubs by ensuring that “the sport club is open for everyone without requiring performance”. At the beginning, this became a category; however, a varied meaning emerged concerning the definition of “everyone”. In all the focus group interviews, “everyone” was essentially described as “regardless of gender and age”. However, some sport clubs also included other categorizations, such as sexuality, disability, and ethnicity. This forced us to rethink this categorization. In order to finalize the theory, we sought information in the literature.

## The individual at the centre of a community

**Figure 1.**
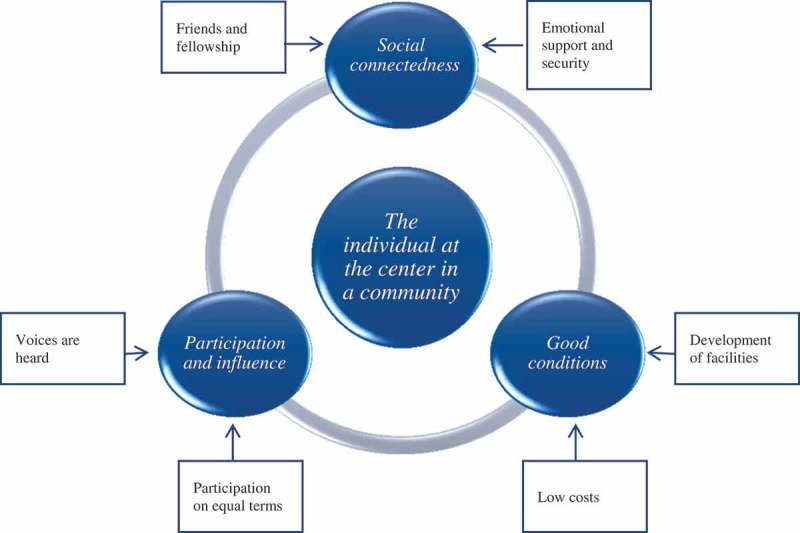
The grounded theory illustrates the social processes of how sport clubs, and particularly coaches, work to encourage young adults to want and be able to remain at the sport clubs.

The core category, *The individual at the centre of a community*, summarizes a process whereby the generated GT contains three main categories, namely (1) *Participation and influence*, (2) *Social connectedness*, and (3) *Good conditions*. Additionally, the main categories contain six subcategories revealing social processes of how sport clubs, and particularly coaches, work to encourage young adults to want and be able to remain at the sport clubs. The core category was that central values in the sport clubs show that the coaches pay attention to all young adults, regardless of their background, ambitions, and skills. Their needs and interests are given higher priority than those of the individual coaches and sport clubs. The theory is presented in Figure 1. Indeed, rather than showing a final act of processes that retain young adults at sport clubs, our theory instead reveals that the sport club culture is continually constructed and re-constructed within complex social and power dynamics, and cannot definitively be achieved. Below is a report of the findings.

### Participation and influence

#### Voices are heard

Participation and the possibility to influence was an important issue to keep the participants at the clubs. The stories from the young adults revealed that their voices have been heard, in that they say what they think or make requests or suggestions concerning their clubs. It was not just about presenting comments and requests for training and competitions, but also regarding establishing start-up meetings in which coaches listen to the young adults and their views about competitions the club should arrange, and tasks the young adults can participate in and take responsibility for. The young adults also described the importance of having an open atmosphere at the sport clubs, in which there is a constant dialogue between coaches and young adults, as well as the importance of coaches listening to their opinions before making decisions. Below is an example from a FG in which the following question was asked: what opportunities do you have to affect the activities in your sport club?

- Great.
- Very great.
- They do have membership meetings, so that you can say what’s on your mind … all ages.
- We have a board, but they make very few decisions themselves. They hardly ever make decisions by themselves … instead they ask at meetings, the homepage, and on Facebook and so … you talk to each other so they are not sitting there and deciding everything.
- But do you feel that you are able to talk?
- Yes, when you have something to say.
- Then you say it … and it feels like people are also listening.

Although it was claimed in all FGs that young adults could make their voices heard, some young adults asserted that they did not know what they could actually influence at the club, or what they would like to influence. Some young adults who played floorball said that they wanted the club to give priority to the elite teams at their club; at the same time, they understood the club values offering equal opportunities and equal economic support to all teams. The coaches of the club also confirmed that some members would prefer the club to differentiate between the best teams and other teams, but the club had a clear policy that stated that all teams should have the same opportunities, regardless of ambition, skills, or level.

The coaches in all the focus groups asserted that they listen to the young adults, and consider their desires in order to meet their needs. Although the analysis did not demonstrate that the clubs had implemented some form of youth council, it appeared that these organizations were trying to encourage all members to come up with new ideas. The coaches also said that, as leaders, it is their responsibility to create as good a relationship with the young adults as possible, as this allows all the young adults to feel comfortable when expressing their opinions.

#### Participating on equal terms

It was asserted in all the FGs that there were no expectations regarding performance, as this was not a primary objective at the clubs. The coaches declared that even when the young adults trained together, they were treated as individuals, and the coaches tried to incorporate individual aspects of learning when implementing exercises and teaching skills. However, some young adults in floorball expressed concern that players would not remain at the club if extra resources were not spent on the best teams. Regardless of ambition and skills, the young adults said that learning new things, developing different skills, and succeeding in something in which they have invested a lot of time was fun and motivating. In the following dialogue, one can see how everybody, regardless of ambition, is allowed to participate in training sessions.

- There are all levels. Those who go for world championships and those who come here just to have fun … and just want to keep up their fitness.
- I went from doing it a lot … I wanted to compete and develop, but now, it is more that I think it is nice to just go out to the forest and move around.
- It is diverse … different levels. But there are several different levels.
- There is always someone to train with.

All the coaches said that this focus on the right to participate, regardless of ambition, is important in order to get young adults to remain at the clubs and to develop a mutual understanding of one another. The young adults said that they have busy lives, which means that during different periods they neither can nor want to spend much time on sports. Therefore, according to the coaches, the sport clubs are careful to create activities that take the life situations of their members into account. As a result, there is no requirement that the young adults must train for a certain number of hours or achieve a specific result.

All participants in the FGs revealed that the clubs have activities to suit all ages, and it is considered positive when members of different ages participate together. As a contrast, one club had restrictions on the participation of the youngest members, but this was due to a security risk involving the handling of weapons. All the FGs also revealed that everyone is included and has the right to participate, regardless of gender, which was viewed as important. Although gender was not equally distributed among the members of two of the clubs, these two organizations stated that they had just taken action to attend to these conditions. These two sport clubs might exclude young men, and young women, who do not identify with dominant gender norms. While all clubs had activities for both sexes, the young adults clearly stated that, within the individual sports, it was good that their training was mixed and not divided by gender, as this was fun and created a larger community at the sport club. In several of the clubs, the coaches stated that they are actively working with LGBT issues. The young adults said that everyone is welcome at the clubs, regardless of sexual orientation, and that they considered this to be a non-issue. In one FG, the young adults discussed the following:

- There is someone on our board … and s/he is gay. Several in our club are gay … but for us … it doesn’t matter.
- For us, no one really cares.
- We do not think about it … do you understand what I am saying? It feels weird talking about it, because all of us here are human beings.

According to both the young adults and the coaches, everyone is welcome to their sport clubs, regardless of socioeconomic status. They also emphasized that everyone was treated equally. Clubs that are situated in multicultural, low socioeconomic environments demonstrated greater ethnic diversity than other clubs, and the young adults at these clubs did not feel that they were treated differently because of their socioeconomic status. One of the young adults said:

- No, no, there is no difference … who you are. There is no status at all. You do not have status outside of this club, it doesn’t matter. You can be from a rich family, or a poor one. It doesn’t matter … ethnicity or background … it doesn’t matter. Well, I am just saying that I have been here … and see that it is a fine club. I believe that you can look at us, you can go down there and look at the coaches … it is multicultural.

Nevertheless, the young adults stated that they were aware that their club reflects the local population; that is, the club was located in an area that reflected a certain socioeconomic status. In one of the FGs that represented a club that was located in an area with a higher socioeconomic status, this was highlighted in the following way:

- Everyone who rides here has a good socioeconomic status, then … there is a certain group from society that is here … sort of. Our area is one of the districts that have a higher standard. Just because it is situated here makes a great difference.
- Yes, since the typical member already lives in the area.

In order to promote social inclusion in these sport clubs, i.e., allowing more young people to attend and remain, structural conditions such as indirect costs of sports participation, for instance clothing and equipment, must be kept very low. Otherwise, individuals would be excluded on financial grounds. Coaches from several clubs also said that it is difficult to recruit members from socioeconomic environments outside where the club is geographically located. The coaches insisted that a club located in an environment that is characterized by weaker socioeconomic conditions does not struggle to retain young adults more than any other club.

In all the FGs, the opinion was expressed that the clubs are characterized by an open-minded attitude toward human differences. One of the coaches, who belonged to an ethnically diverse club, highlighted the following:

- Tolerance, I would say, is a central thing. You may look as you please, be young or old, tall or short, you decide. No one … everything is accepted and that is … that is the core … that is what we built the club on in the first place. That it is … it is really a high ceiling, you can worship any religion …everyone is allowed. Sort of … everyone can … if you wear a veil or not, it does not matter and I believe that’s the strength. And you feel it pretty quickly. When you come in. You feel it … you get accepted very fast.

Among both the coaches and the young adults who belonged to clubs where there was no ethnic diversity, the perception was that their sport was not particularly known or popular among young adults who were not native Swedes. At the same time, they argued that their club existed in an area that did not have much ethnic diversity. In most cases, these clubs did not actively work to create an ethnically diverse club, but they did declare that everybody was welcome to the club, regardless of ethnicity. These coaches and young adults acknowledged social injustice, but they did not openly express solidarity with excluded young people or criticize the absence of a more “inclusionary” sport club.

Furthermore, there were clubs that did not have any young adults or other members with disabilities. At these clubs, the coaches said that disability was not something that was within their core business yet, but they were working on it. However, other coaches did have experiences with young adults with disabilities at their clubs. They made it clear that their core business had an inclusive approach, and that the club was based on the differences of their members. They pointed out that, for example, football clubs do not have an inclusive approach.

### Social connectedness

#### Friends and fellowship

Regardless of whether they were engaged in a team sport or an individual sport, the young adults reported that they felt a social connectedness at their sport club. Among other things, fellowship and affinity with peers were an important reason for young adults to continue attending their sport clubs. The discussions among the coaches, regardless of club and sport, revealed that they strived to create a meeting place where everyone experiences “a club feeling”, has fun, and feels equally welcome. They explained that when everyone feels welcome and gets along, this club feeling is created. A similar picture emerged when speaking with the young adults.

The young adults reported having deeper social relationships at their sport clubs. They found it enjoyable to hang out with friends at their club, and most of the young adults had known each other for a long time. All the young adults explained that their motivation to continue with their specific sport was due to several reasons, such as enjoyment (the sport itself and friends), personal development, and physical as well as psychological well-being. However, one of the primary reasons was because of friends and fellowship. The following example is from a team sport:

- Because it is fun to see friends ….
- It is an enjoyable sport.
- Very social on my part, anyway.
- Mostly social, anyway. That is true for both the men’s team and the women’s team. We are such a tight group … everyone has grown up with one another. A great gang … everyone knows one another.
- Many are from the same area and it feels as if everyone knew each other from before.
- And it is fun to move around, that’s part of the deal.
- Then the club is very good … to get people to hang around, it feels like that.
- There is a good unity in the whole club. From junior up to senior teams.

In the individual sports, it was also obvious that friends and friendships were an important key for members to stay at the club:

- The truth is that I am happy.
- Me too, actually. Just now I have a lot to study, but I am still here training.
- It is the fellowship that makes you happy when you come.

In a couple of the FGs, it was said that the club was a meeting place where young adults can meet different types of people, everyone is respected, and social connectedness exists, despite the differences. The coaches said that adolescents and young adults develop as individuals by meeting various individuals of different ages and backgrounds. The coaches of these clubs are trying to nurture young adults, which encourages different groups at the club to socialize and respect each other. Both coaches and young adults at some of the clubs indicated that it is important to follow the norms and rules of the clubs, as this fosters feelings of social connectedness and belonging. At one of the clubs, the young adults discussed the notion that the club has made it clear that it is important to have the right attitude and that everyone is equal.

#### Emotional support and security

The stories with the coaches painted a picture of the training environment as supportive for the young adults. The coaches stated that they aimed to create a training environment in which the participants receive support in their sports, from the coaches as well as from each other. According to the coaches, all young adults are offered the opportunity to participate in some form of leadership education or to help with coaching activities. One of the young adults declared:

- Precisely (you help one another), and coach one another! We are always coaching each other. When someone wins a match, I feel, “Yes, I helped to coach him”!

The young adults said they have good relations with the coaches, and that the coaches support everyone, not just some members, which is important in order to feel connected and to want to stay at the club. It is, therefore, of great importance to receive emotional support and be acknowledged by the coach. In the FGs with young adults, participants also explained that their training environment is perceived as safe for newcomers to the club, and that everyone must welcome newcomers.

Besides the role that coaches play in helping young adults in their sports and with their sporting goals, some of the coaches stated that they also support the young adults socially, both inside and outside the sporting context. They want to create a secure environment for the young adults so they do not need to worry about their jobs or schoolwork. The young adults felt that this contributed to the feelings of social connectedness and security. They articulated the view that the coaches and friends at their sport club could be regarded as their second family. An example of this was expressed in one of the FGs:

- I recognize it. At one time, something had happened to me, then I was … I came here, but I could not workout. I had my mind somewhere else. Then both coaches came, and everyone else talked to me.
- After that episode, I felt directly that … this is my second family. This is my family. Everyone is there for you, no matter what. They had me think of other things and encouraged me to keep going.

The coaches in the FGs positioned themselves as being respectful coaches who emphasized various aspects regarding the importance of feeling good, i.e., promoting well-being. In some of the clubs, whole families participated in training camps or competitions, and the young adults felt that their sport was a family matter. This cohesion also helped many young adults to remain at the club.

### Good conditions

#### Low costs

The coaches stated that they aim to foster good conditions at their clubs, which, for example, include low costs and low membership fees in order to encourage members to stay as long as possible. This is made possible by both governmental and municipal contributions. The coaches declared that sports in general can cost quite a lot, and that membership fees can make a difference. Therefore, they want to keep the membership fee as low as possible. The coaches further explained that they are actively working to provide low costs for competitions and training, and that some of the clubs offer free memberships to young adults because economic conditions should never affect the ability to train or compete. The following discussion illustrates this:

- We need all the contributions we can get. Otherwise, we lose the members we need to keep the most … because this is probably the only sport you can choose if you are poor.
- And we will not raise any costs just because we switched facilities and the rent is 10 times as high. That is our strength. It is cheap and you can afford it … everyone can afford to be part of it. That is our motto. It will probably remain as such.

Even the young adults discussed the perception that clubs aimed to support them by keeping costs down:

- They have cheap gloves, which is good. In the city it is SEK 700, and they are 300 here. Then dental protection … you know, the ones for 500 … you get a lot to take home. Then if it is something that is missing, they fix it.

To reduce the cost for the young adults, the clubs even offer to lend materials or equipment, both the clubs with high costs and ones with low costs. The coaches also revealed that the clubs are engaged in voluntary work as an alternative to sports associations that are commercially or professionally run.

#### Development of facilities

Development of facilities in the form of the club sites was also important in order for all members to convene and feel welcomed by the organization. The coaches also said that there is an ongoing development of facilities in order to meet the needs of all members. This allows the facilities to cater to everyone, regardless of the frequency at which they train, and encourages the members to engage in activities aside from training. In several of the FGs, high-quality and proper facilities persuaded members to stay at the club. The young adults explained that they were very satisfied with the fact that the facilities have evolved a lot over time. One of the coaches reported that she felt that the young adults really appreciate and even feel pride when they receive new facilities, instead of using dilapidated ones:

- I have the impression that they get the feeling, “You really mean something, and of course you should have a facility without mould!” Because we actually trained in a facility with mould. So, I believe that it has an incredible importance that they feel like they are part of a bigger picture … that they are valued in some sense. Because now they come and I feel like they are a little proud. A good facility, a good club with a good reputation. It is a little like a whole package. Plus, I believe that it will attract even more people.

Nevertheless, the view was also expressed that the costs of the facilities create tension at the clubs, as many hours of voluntary work are also required to manage the amenities.

### Reflections on the findings

This study has developed the first theory of a sport club culture to retain young adults, as experienced by young adults and coaches. The core category, *The individual at the centre of a community*, shows central values at the sport clubs in that the coaches pay attention to the needs and interests of the young adult members, regardless of their background, ambitions, and skills.

The needs and interests of young adults are also given higher priority than those of the coaches and sport clubs. Thus, the findings show that the voices of the young adults are heard. According to Trondman (2011), this can be considered as a part of the participatory aspects in the SSC policy programme, “Sport Wants (Idrotten vill)”. However, in contrast to Trondman (2005), it is not only the young adults who take part in sports in accordance with a performance-oriented core logic who are given the space to be heard and opportunities to influence the organization. These findings are in line with the two parallel missions that the Swedish sports movement is based on: (1) democratic principles stating that everyone has a right to influence, be involved, and participate (“club fosterage”), and (2) selection logic to create the best athletes (“competitive fosterage”) (Peterson, 2008). Often, these two missions cannot work in parallel with each other, but in this study these ideas work well in every sport club, except for the team sport club (Peterson, 2008).

The findings in this study also show that the sport clubs represent a diversity approach based on the desire to include all young adults, independent of different ambitions, skills, gender, age, and sexuality. Accordingly, the findings in this study are in line with the SSC (2009) policy programme, “Sport Wants (Idrotten vill)”, which asserts that sporting activities should be open to and provide equal opportunities for everyone, and organized in accordance with the needs and interests of young people. However, not all of the sport clubs in this study represent young adults with some form of a disability, different socioeconomic positions, or ethnicities. This depends on the setting in which the sport clubs are situated, as the clubs have found it difficult to recruit members from other neighbourhoods or areas. This also seems to be because these sport clubs were not sufficiently interested and engaged in being a more “inclusionary” sport club. Additionally, some of the sport clubs have not developed activities that include individuals with disabilities yet. The reduction of diversity to a few of its many dimensions can also reflect the struggle that many clubs have expressed to operationalize diverse populations in the real world (Spaij et al., 2014). Even if a sport club believes in diversity as an institutional value, there can still be conflicts or disagreements over which types of diversity are desirable (Spaaij et al., 2014). It seems that diversity in the sport clubs was interpreted through the lens of white, middle-class, “non-disabled” individuals. Thus, the perspective on intersectionality helped us to visualize that social inclusion and justice orientation differed among the various sport clubs (Mattsson, 2010).

The young adults in this study are involved in sports for different reasons, for example, because sports are enjoyable, the activities have intrinsic value, and the adults want to develop their skills. This is in accordance with previous studies (Goncalves et al., 2011; Light et al., 2013; Lindgren, 2002; Thedin Jakobsson, 2014; Thedin Jakobsson et al., 2014; Ullrich-French & Smith, 2009). However, in this study, the feeling of social connectedness at the sport clubs was expressed as the main reason to remain at the club. This feeling is developed through friends, fellowship, and experiences of security, as well as emotional support from coaches. Although the measurable effect is small, participation in sports has been found to affect feelings of social connectedness (Hoye, Nicholson, & Brown, 2015). It has been shown that minorities can have difficulties when attempting to reach this feeling of social connectedness in a society where there are major socioeconomic gaps (Walseth, 2006). In our study, the clubs represented a homogeneous group of individuals with regard to socioeconomic status, which could have affected the strong feeling of social connectedness. Social connectedness is understood to play a crucial role in identity development, mental health, and well-being (Townsend & McWhirter, 2005), in addition to feelings of meaningfulness (Thedin Jakobsson, 2014; Thedin Jacobsson et al., 2014). Recent findings also show that the social environment in and through sports enables youth to gain experiences that will contribute to positive youth development (PYD) outcomes (i.e., Côté, Turnidge, & Evans, 2014; Flett, Gould, Griffes, & Lauer, 2013; Holt et al., 2017). Therefore, it might be reasonable to consider that the young adults in this study might have had a PYD climate (with coaches, peers, families, and other members) that contributed to the fact that they have continued to participate in their sport clubs.

It is important to recognize the value placed on the emotional support that coaches give the young adults, both inside and outside the sports environment. The young adults felt recognized, which encouraged the club to be viewed as a safety zone where they felt at home. The coaches appear to practise a form of caring which produces and reinforces the feelings of security, trust, and social connectedness (e.g., Noddings, 2003). The emotional support from the coaches and their friends at the club seems to contribute to the impression of the sport club as another family. A literature review reveals similar findings, i.e., there are coaches who offer young people social support in the form of material, informational, and emotional support. Moreover, various coaches, parents, and peers appear to play important roles concerning emotional support for young people (Sheridan, Coffee, & Lavallee, 2014). Support, both inside and outside the sporting context, has also been proven to be the most common reason for a feeling of connectedness in a Norwegian study among young Muslim women with immigrant backgrounds (Walseth, 2006).

Another prerequisite to keep as many young adults as long as possible in sport clubs is, according to the coaches, the decision of the clubs to offer low costs, such as reduced member fees and training fees, as well as the possibility of borrowing sports equipment. This means that the willingness of these clubs to retain young adults is not conditional on their financial resources, compared to the appearance of similar situations for various other sports (see, for example, Robertsson & Hvenmark, 2015). However, if the financial contributions of the clubs are reduced, the participation among young adults with a low socioeconomic status may be affected. The financial contributions can, therefore, only be seen as a temporary assurance that the structural conditions are favourable for all young adults. Another prerequisite to keep costs down in the clubs is extensive voluntary labour (Robertsson & Hvenmark, 2015). This did not emerge as a specific perspective in our study, although it is highly probable. In this context, it is also worth noticing that while club facilities are a major expense for the clubs, they are an important investment and allow members to convene and feel at home. It is, however, important to note that relatively extensive financial resources and grants are required to operate a sport club in which everyone can be accommodated, in addition to operations largely executed on a nonprofit basis.

## Methodological considerations

Fifty-five participants are not a small sample in a qualitative study, but only one team sport was found and, thus, participated in the study. The transferability of the information to populations beyond the studied group is limited. Fortunately, empirical processes built into CGT, such as theoretical sampling and memoing, allow for foundational theory development, despite a small sample. Other research decisions—the explicit transparency about CGT research methods, active management of reflexivity, and inclusion of verbatim quotations—addressed issues of trustworthiness. Furthermore, in order not to over-interpret the information, we included data from both young adults and coaches. The fact that there was a direct question about what certain sport clubs do to encourage youth and young adults to remain in their sport clubs can be regarded, from a CGT perspective, as a way to increase trustworthiness (Charmaz, 2006).

## Conclusion

This study offers unique insights into what sport clubs, specifically the coaches, are doing in order to retain young adults at their clubs. The coaches *put the individual at the centre of a community* and pay attention to the needs and interests of all the young adults, regardless of their background, ambitions, and skills. A plausible conclusion is that the identified key components, namely *participation and influence*, s*ocial connectedness*, and *good conditions*, have the greatest impact in encouraging young adults to remain at their clubs. However, while the idea of a moral imperative to provide for diversity was not directly absent in the discussions with both the coaches and young adults, most of the diversity approaches seemed to be based on ambition and skills, gender, age, and sexuality. Diversity in sport clubs was interpreted through the lens of white, middle-class, “non-disabled” individuals. This grounded theory offers a tool to understand how the social processes are running in parallel. Concepts from the theory could be used by sport clubs, especially sport coaches, that want to support adolescents and young adults in their path to adulthood by empowering and enabling them to find joy, maximize their participation, and improve their health and well-being. Taking the insights from this study into consideration, more research is warranted to test different team sports and sport clubs that represent different grades of social inclusion.
